# Influence of cholesterol/caveolin-1/caveolae homeostasis on membrane properties and substrate adhesion characteristics of adult human mesenchymal stem cells

**DOI:** 10.1186/s13287-018-0830-4

**Published:** 2018-04-03

**Authors:** Jihee Sohn, Hang Lin, Madalyn Rose Fritch, Rocky S. Tuan

**Affiliations:** 1 Center for Cellular and Molecular Engineering, Department of Orthopaedic Surgery, University of Pittsburgh School of Medicine, 450 Technology Drive, Room 221, Pittsburgh, PA 15219 USA; 2 Department of Bioengineering, Swanson School of Engineering, University of Pittsburgh, Pittsburgh, PA 15219 USA

**Keywords:** Mesenchymal stem cells, Caveolin-1, Caveolae, Cholesterol, Membrane properties, Membrane fluidity, Cell adhesion

## Abstract

**Background:**

Adult mesenchymal stem cells (MSCs) are an important resource for tissue growth, repair, and regeneration. To utilize MSCs more effectively, a clear understanding of how they react to environmental cues is essential. Currently, relatively little is known about how the composition of the plasma membranes affects stem cell phenotype and properties. The presence of lipid molecules, including cholesterol in particular, in the plasma membrane plays a crucial role in regulating a variety of physiological processes in cells. In this study, we examined the effects of perturbations in cholesterol/caveolin-1 (CAV-1)/caveolae homeostasis on the membrane properties and adhesive characteristics of MSCs. Findings from this study will contribute to the understanding of how cholesterol/CAV-1/caveolae regulates aspects of the cell membrane important to cell adhesion, substrate sensing, and microenvironment interaction.

**Methods:**

We generated five experimental MSC groups: 1) untreated MSCs; 2) cholesterol-depleted MSCs; 3) cholesterol-supplemented MSCs; 4) MSCs transfected with control, nonspecific small interfering (si)RNA; and 5) MSCs transfected with CAV-1 siRNA. Each cell group was analyzed for perturbation of cholesterol status and CAV-1 expression by performing Amplex Red cholesterol assay, filipin fluorescence staining, and real-time polymerase chain reaction (PCR). The membrane fluidity in the five experimental cell groups were measured using pyrene fluorescence probe staining followed by FACS analysis. Cell adhesion to collagen and fibronectin as well as cell surface integrin expression were examined.

**Results:**

Cholesterol supplementation to MSCs increased membrane cholesterol, and resulted in decreased membrane fluidity and localization of elevated numbers of caveolae and CAV-1 to the cell membrane. These cells showed increased expression of α1, α4, and β1 integrins, and exhibited higher adhesion rates to fibronectin and collagen. Conversely, knockdown of CAV-1 expression or cholesterol depletion on MSCs caused a parallel decrease in caveolae content and an increase in membrane fluidity due to decreased delivery of cholesterol to the cell membrane. Cells with depleted CAV-1 expression showed decreased cell surface integrin expression and slower adhesion to different substrates.

**Conclusions:**

Our results demonstrate that perturbations in cholesterol/CAV-1 levels significantly affect the membrane properties of MSCs. These findings suggest that modification of membrane cholesterol and/or CAV-1 and caveolae may be used to manipulate the biological activities of MSCs.

**Electronic supplementary material:**

The online version of this article (10.1186/s13287-018-0830-4) contains supplementary material, which is available to authorized users.

## Background

Regenerative medicine aims to re-establish lost tissue function within complex *in vivo* environments. Endogenous or exogenous stem cells, such as adult mesenchymal stem cells (MSCs), are an attractive cell source to utilize for effective restoration of tissue function by cell-driven tissue synthesis. MSCs possess the ability to proliferate and differentiate into different cell types, including osteoblasts, adipocytes, and chondrocytes, dependent on their environmental conditions [1–3]. The attractiveness of MSCs stems from their multipotent differentiation potential and relative ease of isolation, in addition to their immunomodulatory properties and release of trophic factors [4, 5].

A landmark discovery in stem cell-environment interactions was made by Engler et al. [6] who reported that the stiffness of two-dimensional (2D) adhesion substrates can determine the differentiation of MSCs *in vitro*. These investigators showed that MSCs differentiate into neurogenic, myogenic, and osteogenic phenotypes on substrates that have elasticity values similar to those of brain, muscle, and bone tissue, respectively [6]. This mechanosensory behavior is a potentially critical parameter in the application of MSCs for tissue engineering. Therefore, to utilize MSCs more effectively, a clear understanding of how they react to environmental cues is essential. While many studies have focused on derivation and molecular regulation of stem cells, relatively little is known about the composition of the stem cell membrane, the organization of which can critically affect cell responses to external stimuli.

Cell membranes represent not only important cellular barriers, but also first-contact structures of the MSCs with their extracellular matrix (ECM). Lipid rafts are cell membrane microdomains that influence the organization of cell signaling molecules [7]. The organization of signaling molecules in MSC membrane lipid rafts likely plays an important role in governing stem cell phenotype and responsiveness to external stimuli. Lipid rafts of cell membranes fall into two broad categories, noncaveolar lipid rafts and caveolae, based on the absence or presence of caveolin proteins, respectively [8, 9].

Caveolae are flask-shaped plasma membrane invaginations that are sphingolipid and cholesterol-rich. The defining feature of caveolae is the presence of the protein caveolin [10, 11]. Caveolin-1 (CAV-1), a scaffolding protein, is the main protein component of caveolae and has been implicated in numerous cellular functions, including cholesterol export, endocytosis, and regulation and organization of cell signaling molecules [11, 12]. Most importantly, CAV-1 binds to cholesterol and drives caveolae formation [13, 14]. Previous studies performed with human skin fibroblasts reported that intracellular cholesterol levels are closely related to CAV-1/caveolae expression and activity. Intracellular accumulation of cholesterol promotes CAV-1 expression, and CAV-1 directs the export of cholesterol to caveolae in response to increased intracellular cholesterol uptake or synthesis [15, 16]. Together, these studies strongly suggest that CAV-1 may also be involved in regulating organization and the distribution of caveolae and lipid membrane rafts. Furthermore, CAV-1 expression may be regulated by cholesterol and/or vice versa. However, in stem cells, CAV-1/caveolae and membrane cholesterol regulation and their functional relationships are incompletely understood and further investigation is required, particularly in MSCs.

Cholesterol is a critical component of the plasma membrane and plays a key role in determining the physical properties of the lipid bilayer [17]. Thus, modification of cell membrane cholesterol, CAV-1, and caveolae contents should affect membrane properties such as fluidity. In fact, cholesterol or lipid integration into the cell membrane has been reported to decrease membrane fluidity in HEK 293 T cells [18]. In addition, perturbation in membrane cholesterol, CAV-1, and caveolae levels may affect the availability of cell surface adhesion receptors and integrin expression [19], which further affect cell adhesion potentials. Integrins function as a major class of transmembrane cell substrate receptors, consisting of heterodimers of α and β subunits that bind to epitopes on different ECM substrates with different degrees of stability [20, 21]. As the principal adhesive ECM receptors in the cell membrane, the activity of integrins plays a vital role in MSC substrate mechanosensing [6, 22]. The stability of cell adhesion to different substrates is dependent on the expression profile of integrins on the cell membrane [23, 24]. Fewer cell surface integrins for a particular substrate decrease the stability of cell adhesions due to less abundant/stable interactions. Additionally, amplified contractile forces rupture integrin contacts and increase integrin endocytosis, further decreasing cell adhesion [20]. Interestingly, this integrin internalization occurs via cholesterol-rich, CAV-1-coated caveolae membrane rafts in MSCs on 2D substrates [25]. Several studies have shown that the endocytic mechanism that regulates β1 integrin is mediated by lipid rafts, and that down- or upregulating the lipid raft protein, CAV-1, significantly changes β1 integrin endocytosis [25–27]. In addition, CAV-1 can promote activation of signaling proteins in focal adhesions, as well as downregulate it by driving the caveolar endocytosis of focal adhesion proteins [19, 28, 29], which can further affect integrin function and activity. These studies suggest that if the expression and activity of CAV-1/caveolae is intimately linked with membrane cholesterol status in MSCs, perturbing cholesterol/CAV-1/caveolae in MSCs should affect membrane fluidity, substrate adhesion rate, and cell surface integrin expression. Moreover, the activity of the cholesterol/CAV-1/caveolae system may influence cell mechanosensing, and cholesterol and CAV-1 may be targets for manipulation of MSC responses to physical substrates.

This study was designed to test the effects of perturbations in cholesterol/CAV-1/caveolae homeostasis on human bone marrow-derived MSC membrane properties and adhesive characteristics. To determine the functional relationship of cholesterol and CAV-1/caveolae and their potential influence on MSC activities, we generated five experimental MSC groups: 1) untreated cells serving as control; 2) cholesterol-depleted MSCs; 3) cholesterol-supplemented MSCs; 4) MSCs transfected with control small interfering (si)RNA; and 5) MSCs transfected with CAV-1 siRNA. Each cell group generated was analyzed for perturbations of cholesterol status and CAV-1 expression. Our results showed that perturbations in cholesterol/CAV-1/caveolae levels affect the membrane properties of MSCs. Cholesterol supplementation in the culture medium resulted in increased cell membrane cholesterol content, thus localizing elevated numbers of caveolae and CAV-1 to the cell membrane and, as a result, these cells had decreased membrane fluidity. These cells showed increased expression of α1, α4, and β1 integrins and thus had higher adhesion rates to fibronectin and collagen. Conversely, knockdown of CAV-1 expression or cholesterol depletion on MSCs caused a parallel decrease in the number of cell surface caveolae and an increase in membrane fluidity due to decreased delivery of cholesterol to the cell membrane. Cells with depleted CAV-1 expression showed decreased cell surface integrin expression and demonstrated slow adhesion to different substrates. Taken together, our findings suggest that treatments that increase or decrease membrane cholesterol and/or CAV-1 and caveolae in MSCs may be used to manipulate the biological activities of MSCs.

## Methods

### Tissue collection and harvesting of MSCs

Human bone marrow-derived MSCs were isolated with Institutional Review Board approval (University of Washington, Seattle, WA) from the femoral heads of patients undergoing hip arthroplasty using a standard plastic adhesion protocol. Cells flushed from the bone marrow were pelleted and resuspended in isolation medium (ISM) (α-minimum essential medium (MEM) + 1× antibiotic-antimycotic + 10% MSC qualified fetal bovine serum (FBS; all Gibco/Thermo Fisher Scientific, Waltham, MA, USA) + 1 ng/ml fibroblast growth factor (FGF)2 (R&D Systems, Minneapolis, MN)), seeded into T150 flasks (Corning Inc., Corning, NY, USA) and incubated for 3–4 days at 37 °C under 5% CO_2_, then washed twice with phosphate-buffered saline (PBS; pH 7.4) and incubated in fresh isolated medium. When colonies reached 80% confluence, cells were harvested with trypsin/EDTA and resuspended at 1 × 10^6^ cells/T150 flask in ISM. Medium was changed every 3–4 days when cells reached 80–90% confluency, at which time cells were either frozen or passaged. MSC isolates were routinely characterized with respect to multilineage differentiation capabilities as described previously [30]. Multiple donor MSCs were pooled (Additional file 1: Table S1) and used in this study.

### Cell culture and treatments

#### Cell culture

For the experiments, MSCs were expanded in proliferation medium (PM) containing high-glucose Dulbecco’s modified Eagle’s medium (DMEM) containing l-glutamine and sodium pyruvate + 1× antibiotic-Antimycotic + 10% MSC qualified FBS (all Gibco/Thermo Fisher Scientific, Waltham, MA, USA) at 37 °C under 5% CO_2_. PM was changed every 3–4 days until cells reached 70–80% confluence, at which time cells were seeded for experiments.

#### Cell membrane cholesterol manipulation

Disruption to all cholesterol membrane rafts was achieved by 60-min preincubation with methyl-β-cyclodextrin (MβCD; Sigma-Aldrich) in DMEM used at a final concentration of 10 mM. As prolonged incubation with MβCD is cytotoxic to MSCs, MβCD was not left on cells beyond the 60-min period. After MβCD pretreatment, cholesterol-depleted cells were cultured in fresh medium containing charcoal stripped FBS (CS-FBS; Gemini Bio-Products, West Sacramento, CA, USA). CS-FBS has been adsorbed with activated carbon, which removes hormones, phospholipids, and free fatty acid fractions in FBS. The removal of serum free fatty acids and lipid-like components has been shown to minimize cell lipid metabolic functions [31].

Cholesterol supplementation was achieved by pretreating MSCs with 10 mM MβCD for 60 min and then incubating cells with fresh medium containing CS-FBS and 100 μM cholesterol-cyclodextrin inclusion complexes. For preparation of the cholesterol-MβCD inclusion complexes (chol-MβCD), small aliquots of cholesterol (Sigma-Aldrich) in methanol-chloroform (2:1 *v*/v) were added to a stirred solution of MβCD (5% *w/v*) in a water bath (80 °C); 1 g MβCD was added to 30 mg cholesterol. The mixtures were stirred at 80 °C until complete dissolution of the initially precipitating steroid. The complex preparations were then freeze-dried and stored at room temperature.

#### siRNA transfection

siRNA transfection was performed as described previously [32]. Briefly, cells were seeded at 20,000 cells/cm^2^, incubated overnight, washed in serum-free and antibiotic-free high-glucose DMEM, and transfected with either 50 nM human CAV1 ON-TARGETplus SMARTpool siRNA or 50 nM nontargeting control siRNA ON-TARGETplus Non-targeting SMARTpool siRNA (all Dharmacon/GE Life Sciences, Lafayette, CO, USA) using DharmaFECT1 transfection Reagent (Dharmacon/GE Life Sciences). DharmaFECT1 and each siRNA were prepared at 10× the final concentration used for transfection in serum-free and antibiotic-free high-glucose DMEM and incubated for 5 min at room temperature. Each siRNA was then mixed 1:1 with the DharmaFECT1, incubated for 20 min at room temperature, diluted 1:5 in antibiotic-free PM, and added to washed MSCs at 0.13 ml/cm^2^. The final volume of DharmaFECT used was 0.08 μl/cm^2^. Twenty-four hours later, the medium was replaced with fresh PM.

### Cell proliferation assay

The number of viable cells in proliferation was assessed using the CellTiter 96 Aqueous One Solution Assay (Promega, Madison, WI), a colorimetric method using 3-(4,5-dimethylthiazol-2-yl)-5-(3-carboxymethoxyphenyl)-2-(4-sulfophenyl)-2H-tetrazolium (MTS). Thirty thousand cells were plated in triplicate on 24-well plates and, 48 h later, CellTiter 96 Aqueous One Solution was added (20 μl/100 μl PM). After incubation for 4 h, absorbance at 490 nm was read using a plate reader.

### Cell membrane cholesterol quantification

Cellular lipids were extracted using a chloroform/methanol (2:1) mixture as described previously [33]. Cell membrane cholesterol levels were quantified with a modified version of a previously described microenzymatic fluorescence assay using the Amplex Red cholesterol assay according to the manufacturer’s protocol (ThermoFisher Scientific, Waltham, MA) [34]. This colorimetric assay is based on the reaction of cholesterol with cholesterol oxidase to yield H_2_O_2_ which can be detected using the Amplex Red reagent. This method includes treatment with cholesterol oxidase to ensure the exclusive determination of free cholesterol present in the plasma membrane, and not of cholesterol esters present in the cytoplasm. Values of total cholesterol concentration were normalized to total cell number.

### mRNA extraction and real-time reverse transcription polymerase chain reaction

Total RNA was obtained from MSCs using Trizol reagent (Invitrogen, Carlsbad, CA) and an RNeasy Mini Kit (Qiagen, Valencia, CA) according to the manufacturer’s instructions. Reverse transcription was performed using a Maxima first strand cDNA synthesis kit (ThermoFisher Scientific) according to the manufacturer’s protocol. Polymerase chain  reaction (PCR) was performed using an SYBR Green PCR master mix (Applied Biosystems, Foster City, CA) on a 7900HT Fast Real-Time PCR machine (Applied Biosystems) as previously described [32]. The primer sequences used were as follows (Life Technologies, Carlsbad, CA): *GAPDH*, forward CAAGGCTGAGAACGGGAAGC, reverse AGGGGGCAGAGATGATGACC; and *CAV-1*, forward GGGCAACATCTACAAGCCCAACAA, reverse CTGATGCACTGAATCTCAATCAGGAA.

### Fluorescent staining

Cells were seeded onto a 24-well glass bottom plate (Cellvis, Mountain View, CA) and fixed with 4% paraformaldehyde (PFA) for 1 h at room temperature. Cells were rinsed with PBS and incubated with 1 ml glycine (1.5 mg /ml PBS) for 10 min at room temperature to quench the PFA. Cells were stained with freshly prepared 50 μg/ml filipin III (Sigma) in PBS for 3 h at room temperature followed by Alexa Fluor 488 Phalloidin (Life Technologies/Molecular Probes, Eugene, OR; 1:50) in PBS. Stained cells were viewed on an Olympus Fluoview 500 confocal microscope (Olympus America, Inc., Center Valley, PA).

### Sucrose gradient subcellular fractionation

Subcellular fractionation was performed according to a previously published method [35]. Briefly, MSCs were homogenized in 2 ml ice-cold 500 mM sodium carbonate, pH 11.0, supplemented with protease and phosphatase inhibitors (1:100, Sigma-Aldrich), sonicated (three times, 20 s each) on ice, mixed 1:1 with 90% sucrose in MBS buffer (50 mM MES, pH 6.5, 0.3 mM NaCl), and placed at the bottom of a 14 × 89 mm^2^ ultracentrifuge tube (Beckman, Palo Alto, CA). After the sucrose gradient was formed by layering 4 ml 35% sucrose in 1:1 MBS:500 mM sodium carbonate, followed by 4 ml 5% sucrose in 1:1 MBS:500 mM sodium carbonate, samples were centrifuged at 39,000 rpm in a Beckman XL-70 Ultracentrifuge (SW40Ti rotor) for 22 h at 4 °C. The contents of the centrifuge tubes were collected in sequential 1-ml fractions from top to bottom to make a total of 12 fractions.

### Western blotting

Cell lysates from 12 fractions of the sucrose gradient subcellular fractionation were used for Western blotting. Proteins were analyzed by SDS-PAGE in a 12% polyacrylamide gel and transferred to polyvinylidene fluoride (PVDF) blots which were blocked in 5% bovine serum albumin (BSA; Sigma) in Tris-buffered saline + 0.05% Tween 20 (TBST), and then incubated overnight at 4 °C with rabbit anti-CAV-1 (1:1000 in TBST+ 2.5% BSA; Abcam). The secondary antibody was Amersham ECL donkey anti-rabbit horseradish peroxidase (HRP)-linked IgG antibody (GE Healthcare UK Limited, Little Chalfont, UK). HRP activity was detected using Super Signal West Dura, Extended Duration Substrate (ThermoScientific™ Pierce™ Protein Biology) and the chemiluminescence reaction was visualized using a FOTO/Analyst FxCCD imaging system (Fotodyne Inc., Hartland, WI, USA).

The intensity of bands on Western blots was measured using grayscale images in Image J software (http://imagej.nih.gov/ij) as described previously [32]. Intensities of CAV-1 bands were normalized to the total protein concentration from the same sample. Total protein concentration before sucrose gradient subcellular fractionation was determined by BCA assay (Pierce Biotechnology, Rockford, IL) according to the manufacturer’s protocol.

### Membrane fluidity

Membrane fluidity of MSCs was measured by flow cytometry using a fluorescent labeling method according to the manufacturer’s protocol (Membrane Fluidity kit, Abcam). Briefly, 200,000 cells were stained at room temperature with the fluorescent lipid reagent containing pyrenedecanoic acid, which exists as either a monomer or an excimer, the latter being formed upon monomer spatial interaction, resulting in a substantial red shift of the emission spectrum of the pyrene probe. A fluorescence-activated cell sorting (FACS) instrument (FACSAria II SORP, Becton Dickenson), equipped with an argon ion laser (360 nm emission, 60 mW output) and bandpass filters of 395 nm (26 nm bandwidth) and 450 nm (50 nm bandwidth), was used for analyzing the fluorescence intensity of labeled cells. Quantitative monitoring of the membrane fluidity was attained by measuring the ratio of monomer to excimer fluorescence.

### Cell adhesion assay

A cell adhesion assay was performed using the Vybrant Cell Adhesion Assay Kit (V-13181, ThermoFisher Scientific) according to the manufacturer’s protocol. Briefly, MSCs were stained with calcein AM solution (final concentration at 5 μM) by incubation at 37°C for 30 min, and 5000 cells were plated into prepared tissue culture plastic, collagen I-coated, or fibronectin-coated 96-well microplates. After incubation at 37 °C for 45 min to 4.5 h, nonadherent calcein-labeled cells were removed by careful washing, and 200 μl PBS was added to each well. The fluorescence intensity of each well was measured using a fluorescein filter set (494 nm absorbance maximum and 517 nm emission maximum). The total fluorescence intensity of plated cells was obtained by omitting the wash steps. The percentage of adhesion was determined by dividing the fluorescence of adherent cells by the total corrected fluorescence of cells added to each microplate well and multiplying by 100%.

### Flow cytometry

One hundred thousand MSCs were collected, washed with PBS containing 2% FBS, centrifuged, and then placed on ice. The cells were then resuspended in 10% FBS in PBS and incubated for 10 min. Phycoerythrin (PE)-conjugated mouse anti-human CD49a (α1 integrin, 559,596, BD), allophycocyanin (APC)-conjugated mouse anti-human CD49d (α4 integrin, 559,881, BD), or APC-conjugated mouse anti-human CD29 (β1 integrin, 559,883, BD) was added to each tube (15 μl/100,000 cells) and incubated for 30 min on ice. The cells were then rinsed in 300 μl cold washing buffer (2% FBS in PBS, 4 °C). The single color antibody was used to optimize fluorescence compensation settings for multicolor analyses of the cell by flow cytometry. Appropriate control mouse Ig isotype was also used for comparison.

### Statistical Analysis

Figures and statistics were generated using GraphPad 7. All data are presented as means and 95% confidence intervals for analyzing correlation of gene expression. Otherwise, mean differences between groups were assessed with a Student’s *t* test. Results are given as the mean ± SD. When more than two groups were analyzed, one-way analysis of variance (ANOVA) was used to calculate statistical significance. *P* values less than 0.05 were considered significant.

## Results

### Generating five experimental groups of MSCS

For all assays, five experimental MSC groups were generated by disrupting either cell membrane cholesterol or CAV-1 mRNA expression in cells. We depleted cholesterol with MβCD, which binds to cholesterol and strips cholesterol from the cell membrane. MSCs were transfected with siRNA specific to CAV-1 to downregulate CAV-1 gene expression [32, 36]. The five experimental groups of MSCs were: 1) untreated MSCs; 2) cholesterol-depleted MSCs (MβCD-MSCs); 3) cholesterol-enriched MSCs (Chol-MSCs); 4) MSCs transfected with control, nonspecific siRNA (si Ctrl-MSCs); and 5) MSCs transfected with CAV-1 siRNA (si CAV-1-MSCs).

### Setting of cholesterol depletion and supplementation conditions

**Fig. 1:**
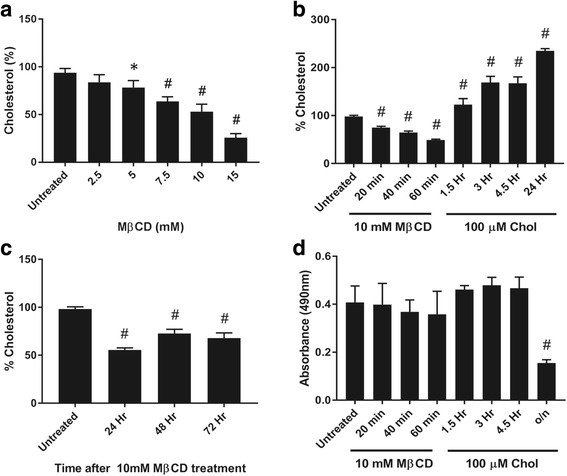
Effects of methyl-β-cyclodextrin (MβCD) and cholesterol (chol)-MβCD treatments on membrane cholesterol level and cell viability of MSCs. **a** Cholesterol level of MSCs after treatment with MβCD for 40 min at the concentrations indicated. Membrane cholesterol levels were measured and compared (untreated cells = 100%). **b** Cholesterol level of MSCs after treatment with 10 mM MβCD or 100 μM chol-MβCD for the time indicated. **c** Time course of cholesterol level of MSCs after initial treatment with 10 mM MβCD for 60 min. After treatment, cells were washed with PBS and incubated in fresh PM containing CS-FBS for the time indicated. **d** Viability of cells, treated as described in Fig. 1b, evaluated by MTS assay. Results are presented as mean ± SD; *n* ≥ 3 independent experiments per cell line per time point. The results showed the effectiveness of MβCD and chol-MβCD in altering MSC membrane cholesterol level without compromising cell viability. *p < 0.05, ^#^*p* < 0.0001, versus untreated group. o/n overnight

MβCD is currently the most commonly used cyclodextrin for cell membrane cholesterol removal and supplementation studies because of its effectiveness at significantly lower concentrations than other cyclodextrins, although the degree of cholesterol depletion varies based on concentrations of MβCD, incubation time, temperature, and cell types [37]. Therefore, initial testing was performed to establish the desired conditions for MβCD-mediated cholesterol depletion from the plasma membrane of human MSCs. Cells were first exposed to different concentrations (2.5–15 mM) of MβCD for 40 min (Fig. 1a); 10 mM and 15 mM MβCD treatments significantly removed membrane cholesterol by 47.0% and 74.3%, respectively. However, the 15 mM MβCD treatment affected cell viability (data not shown). Additional time course testing using 10 mM MβCD showed that a 60-min treatment was able to remove 50.8% cholesterol whilst keeping cell viability almost unchanged (Fig. 1b and d).

To observe membrane cholesterol recovery time after 60 min of 10 mM MβCD treatment, the treated cells were then incubated in fresh PM containing CS-FBS for 48 to 72 h. Membrane cholesterol levels slowly recovered but remained significantly lower than those in untreated cells (Fig. 1c).

For cholesterol enrichment, cells were pretreated with 10 mM MβCD for 60 min and then exposed to cholesterol-MβCD inclusion complexes (chol-MβCD). Compared to untreated MSCs, 100 μM chol-MβCD loading led to a significant increase in membrane cholesterol levels. After 3 h of incubation with chol-MβCD, the membrane cholesterol levels increased by 169.1% without affecting cell viability (Fig. 1b and d). Longer incubation with chol-MβCD decreased cell viability (Fig. 1d).

Based on the results that showed that 10 mM MβCD treatment decreased about 50% of membrane cholesterol and that 100 μM chol-MβCD loading increased about 170% of membrane cholesterol, these conditions were used throughout this study to manipulate membrane cholesterol levels in MSCs.

### Effect on CAV-1 mRNA

**Fig. 2:**
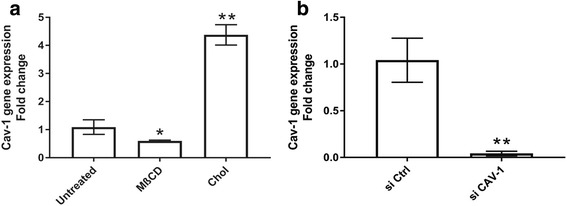
Effects of membrane cholesterol perturbation on CAV-1 mRNA expression. **a** Untreated, cholesterol-depleted MSCs (MβCD), and cholesterol-enriched MSCs (Chol) groups, and **b** control siRNA (si Ctrl) and caveolin-1 siRNA (si CAV-1) treated groups. RNA was extracted from freshly collected MSCs of the five experimental groups and the CAV-1 mRNA level was assessed by real time reverse transcription PCR. The data represent the mean and 95% confidence intervals of the fold-change relative to the untreated or si Ctrl group; *n* = 9. These results showed that CAV-1 mRNA expression was affected by the membrane cholesterol level and confirmed effective siRNA-mediated knockdown of CAV-1 gene expression in MSCs. *p < 0.005, **p < 0.0001, versus untreated or si Ctrl group

The five experimental cell groups were analyzed for the relationship between perturbation of cholesterol status and CAV-1 expression. Quantitative reverse transcription PCR analysis showed that cholesterol enrichment resulted in increased CAV-1 mRNA expression, while cholesterol depletion resulted in decreased CAV-1 mRNA expression, compared with untreated MSCs (Fig. 2a). As expected and based on our previous findings [32, 36], si CAV-1-MSCs showed significantly decreased CAV-1 gene expression (Fig. 2b) compared with si Ctrl-MSCs, lasting up to 8 days after transfection (data not shown). Taken together, these results showed that CAV-1 mRNA levels are responsive to membrane cholesterol levels, suggesting a functional correlation between free membrane cholesterol and CAV-1 gene expression in MSCs.

### Effect on membrane cholesterol levels

**Fig. 3:**
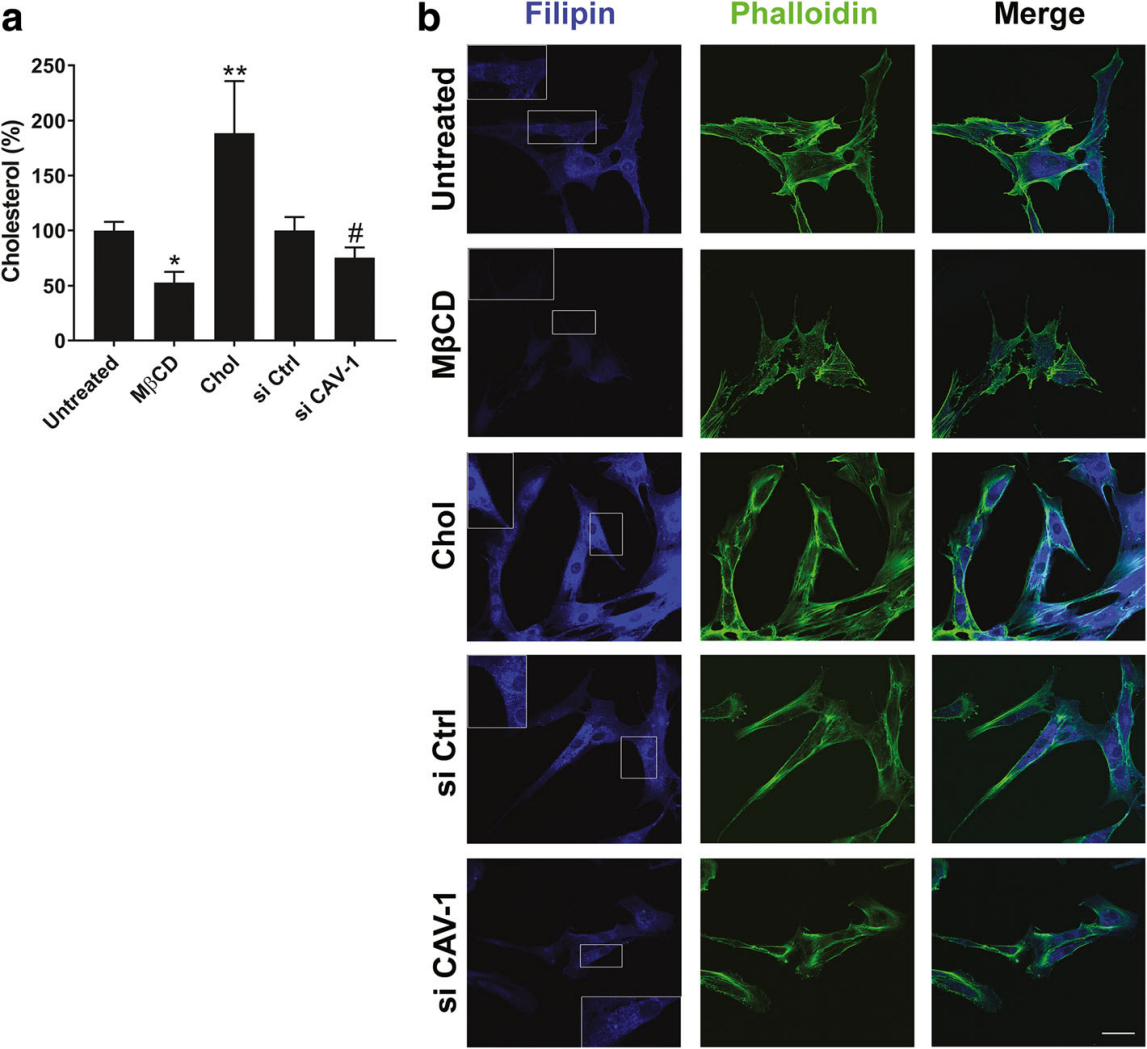
Effects of membrane-specific cholesterol and caveolin-1 (CAV-1) perturbation on membrane cholesterol levels in five MSC experimental groups: untreated MSCs, cholesterol-depleted MSCs (MβCD), cholesterol-enriched MSCs (Chol), MSCs transfected with control, nonspecific siRNA (si Ctrl), and MSCs transfected with CAV-1 siRNA (si CAV-1). **a** Membrane cholesterol content in washed cells was measured and quantified using the Amplex red cholesterol assay and compared with untreated or si Ctrl cells (100%). Data represent mean ± SD; *n* = 9. *p < 0.005, **p < 0.001, versus untreated group; ^#^*p* < 0.05, versus si Ctrl group. **b** Cells were fluorescently stained with filipin for cholesterol (blue) and phalloidin for filamentous actin (green). The scale bar shown represents 50 μm and applies to all panels; (*n* > 4). These results showed that siRNA-mediated knockdown of CAV-1 gene expression significantly decreased membrane cholesterol levels in MSCs

As expected, cell membrane cholesterol analysis of the five MSC experimental groups showed higher cholesterol concentrations in Chol-MSCs (188.7% increase) compared with untreated groups, while MβCD treatment removed 48.1% of membrane cholesterol from MSCs. Knockdown of CAV-1 expression also caused a significant decrease in cell membrane cholesterol (24.8% decrease) compared with si Ctrl-MSCs (Fig. 3a).

Filipin fluorescence staining for free membrane cholesterol was next performed in cultured MSCs, and the staining pattern was observed by confocal microscopy (Fig. 3b). Consistent with the biochemical assays, fluorescence images that represent cellular cholesterol distribution in the five MSC groups indicated increased free membrane cholesterol staining upon cholesterol enrichment, while knockdown of CAV-1 gene and cholesterol depletion caused a decrease in cell membrane cholesterol staining. Furthermore, we observed that CAV-1 siRNA treatment reduced both CAV-1 mRNA levels (Fig. 2b) and membrane free cholesterol (Fig. 3), again supporting a link between membrane cholesterol and CAV-1 expression and level in MSCs.

### Effect on caveolae content

**Fig. 4:**
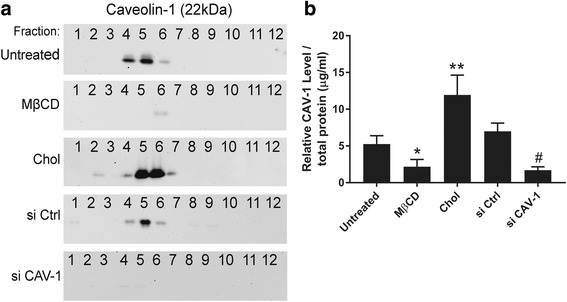
Effects of cholesterol and caveolin-1 (CAV-1) perturbation on caveolae content in MSCs. Sucrose density centrifugation was used to fractionate membrane preparations derived from the five MSC groups: untreated MSCs, cholesterol-depleted MSCs (MβCD), cholesterol-enriched MSCs (Chol), MSCs transfected with control, nonspecific siRNA (si Ctrl), and MSCs transfected with CAV-1 siRNA (si CAV-1). Upon ultracentrifugation, buoyant membrane rafts float to the upper fractions (4–7) of the 12-fraction sucrose gradient and are separated from intracellular fractions (9–12). Caveolae are identified on the basis of CAV-1 content. **a** CAV-1 immunoblotting of the fractions. This result is representative of three, pooled human MSC donor sources tested (Additional file 1: Table S1). **b** CAV-1 signal/total protein amount used for fractionation for each experimental group analyzed by densitometry. Data represent mean ± SD of all experimental replicates, analyzed using NIH ImageJ; *n* > 3. These results indicate that changes in membrane cholesterol levels significantly affect the caveolae content in MSCs. *p < 0.05, **p < 0.001, versus untreated group; ^#^*p* < 0.001, versus si Ctrl group

CAV-1 is the major protein component of caveolae and, in fact, formation and expression of caveolae and CAV-1 are highly dependent on the availability of cholesterol [38]. We therefore analyzed membrane caveolae content in the five experimental MSC groups. Protein samples were harvested and sucrose density gradient subcellular fractionation followed by Western blotting of each fraction for CAV-1 was performed. These results were used as an indication of cell caveolae content [39]. As we showed in a previous study, CAV-1 protein was detected in the buoyant lipid raft containing fractions 4 to 7 obtained by sucrose density gradient fractionation [32] (Fig. 4a). Cholesterol enrichment resulted in increased CAV-1 protein expression, an indication of elevated cell caveolae content. On the other hand, cholesterol depletion and knockdown of CAV-1 gene resulted in decreased CAV-1 protein expression, indicating decreased cell caveolae content (Fig. 4a, b). Taken together, these results provide further evidence that regulation of membrane cholesterol is closely associated with the caveolae, which may be mediated by CAV-1 mRNA.

### Effect on cell membrane fluidity

**Fig. 5:**
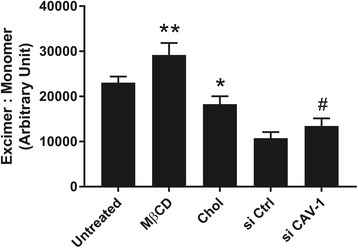
Effects of cholesterol and caveolin-1 (CAV-1) perturbation on membrane fluidity. FACS analysis based on the relative fluorescence intensities of excimer and monomer to assess membrane fluidity was performed on the five MSC experimental groups: untreated MSCs, cholesterol-depleted MSCs (MβCD), cholesterol-enriched MSCs (Chol), MSCs transfected with control, nonspecific siRNA (si Ctrl), and MSCs transfected with CAV-1 siRNA (si CAV-1). Results are presented as mean ± SD; *n* ≥ 3 independent experiments per cell line. The findings show that membrane fluidity is inversely related to membrane cholesterol content, and dependent on the presence of CAV-1. *p < 0.05, **p < 0.001, versus untreated group; ^#^*p* < 0.05, versus si Ctrl group

Perturbation in cell membrane cholesterol is known to affect cell membrane fluidity [18]. Results obtained using the Abcam fluorescence-based pyrene probe showed that cholesterol and CAV-1 depletion increased membrane fluidity, while cholesterol enrichment decreased membrane fluidity in MSCs (Fig. 5).

### Effect on cell adhesive properties

**Fig. 6:**
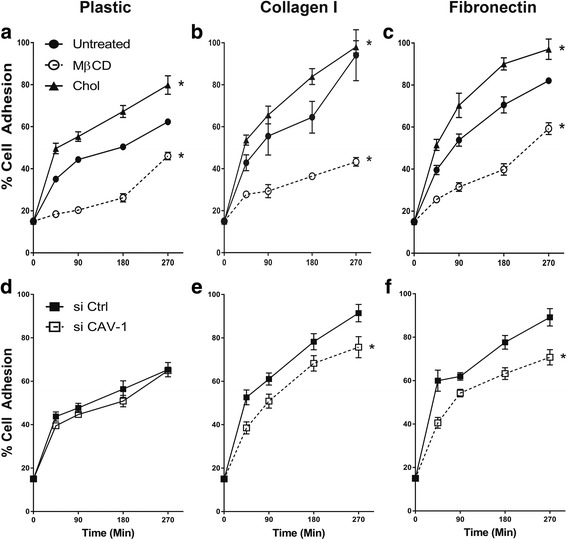
Effects of cholesterol and caveolin-1 (CAV-1) levels on kinetics of MSC adhesion to substrates. **a**,**d** Cell culture plastic (PL); **b**,**e** collagen I (CL); **c**,**f** fibronectin (FN). Adhesion was quantified based on labeled cell fluorescence and expressed as the percentage of adherent cells to total cells added to each microplate well. Results are presented as mean ± SD; *n* = 3 independent experiments per cell line per time point. MSC adhesion to PL, CL, and FN was positively related to membrane cholesterol level (higher adhesion in cholesterol-enriched MSCs (Chol) and lower adhesion in cholesterol-depleted MSCs (MβCD)), while CAV-1 knockdown (si CAV-1; MSCs transfected with CAV-1 siRNA) reduced MSC adhesion to CL and FN. Two-way ANOVA was administered to analyze the data from five MSC groups. *p < 0.0001, versus untreated or si Ctrl group (MSCs transfected with control, nonspecific siRNA)

To assess whether modification of cholesterol content in MSCs could affect MSC adhesion to different substrates, the adhesion kinetics of the five MSC groups to collagen I (CL), fibronectin (FN), and plain cell culture plastic (PL) were measured. As expected, cells adhered more efficiently to CL and FN compared with PL (Fig. 6). The removal of cholesterol from the cell membrane significantly decreased adhesion level (at 45 min: 28%, 26%, 18% for CL, FN, and PL, respectively), while cholesterol enrichment increased cell adhesion (at 45 mins: 54%, 52%, 50% for CL, FN, and PL, respectively) compared with no treatment (at 45 mins: 43%, 40%, 35% for CL, FN, and PL, respectively) (Fig. 6a–c). The elimination or supplementation of cholesterol in MSCs seemed to affect cell adhesion to CL and FN to similar degrees (Fig. 6b, c). Knockdown of CAV-1 gene also decreased adhesion rate of cells to CL and FN (Fig. 6e, f); however, it did not affect the cell adhesion rate on PL (Fig. 6d).

### Effect on cell surface integrin levels

**Fig. 7:**
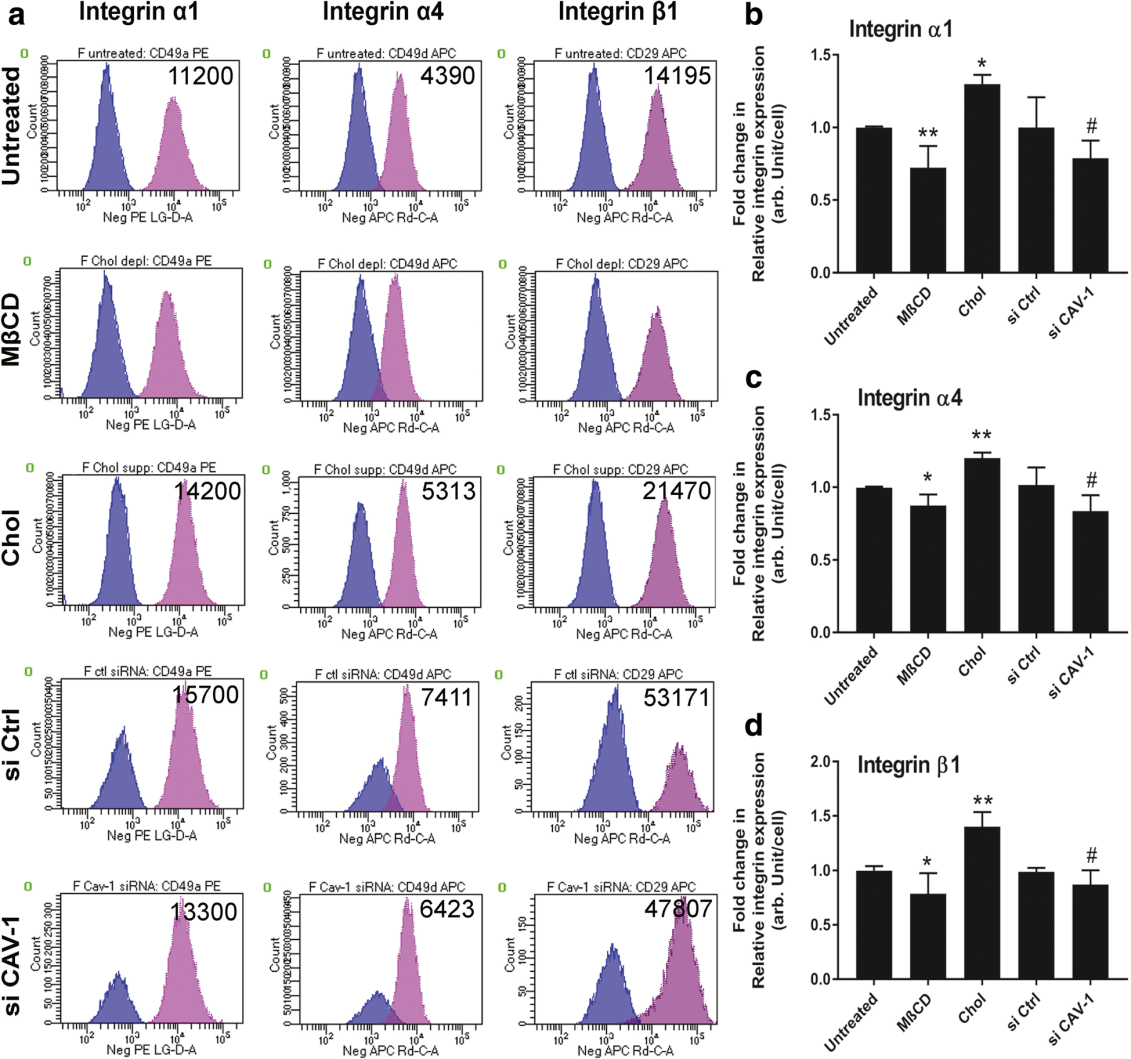
Effects of cholesterol and caveolin-1 (CAV-1) levels on expression of integrins α1, α4, and β1**.** Five MSC experimental groups were analyzed by FACS for integrin α1, α4, and β1 expression: untreated MSCs, cholesterol-depleted MSCs (MβCD), cholesterol-enriched MSCs (Chol), MSCs transfected with control, nonspecific siRNA (si Ctrl), and MSCs transfected with CAV-1 siRNA (si CAV-1). **a** Histograms representing percent cell expression of integrins on cells; mean fluorescence intensity is indicated. **b**–**d** Relative quantitative expression of integrins α1, α4, and β1 on the five MSC groups. Results are presented as mean ± SD; *n* = 3 independent experiments per cell line. These results suggest that, as cell membrane cholesterol content changes, the integrin expression profiles change accordingly. Higher cholesterol concentration on cell membranes resulted in higher levels of integrin expression in MSCs. *p < 0.05, **p < 0.001, versus untreated group; ^#^*p* < 0.05, versus si Ctrl group

The observed increased adhesion rate of MSCs to CL and FN compared with PL (Fig. 6) prompted us to profile the expression of cell surface integrin receptors for collagen and fibronectin, specifically integrins α1, α4, and β1 [40]. Interestingly, flow cytometry results demonstrated that the percentage of integrin α1-, α4-, and β1-positive cells in a population was not affected as a result of cholesterol or CAV-1 gene level changes (Fig. 7a). However, the mean fluorescence intensities of α1, α4, and β1 integrin staining per cell were statistically higher on Chol-MSCs and lower on MβCD-MSCs compared with untreated cells (Fig. 7b–d). The levels of integrin α1, α4, and β1 staining per cell were significantly lower in si CAV-1-MSCs compared to si Ctrl-MSCs (Fig. 7b–d). This finding demonstrates that, although cells with different membrane cholesterol contents expressed α1, α4, and β1 integrins at the same ratio (approximately 95–99%), the level of expression per cell was higher on cholesterol-rich MSCs.

## Discussion

In this study, we present findings showing that perturbations in membrane cholesterol/CAV-1/caveolae homeostasis significantly affect MSC membrane properties and adhesion characteristics. Specialized membrane lipid rafts, called caveolae, are rich in cholesterol and CAV-1 protein [9]. Recently, relations and interactions between CAV-1/caveolae and membrane cholesterol, the main structural components of lipid rafts, are of considerable interest in cell biology. The present study provides evidence for the first time that the two major components of lipid rafts, CAV-1/caveolae and membrane cholesterol, are directly and functionally linked to each other in human MSCs. CAV-1 mRNA levels and caveolae formation are upregulated by free cholesterol loading and downregulated by membrane cholesterol depletion. Furthermore, CAV-1 siRNA-mediated knockdown reduces both CAV-1 mRNA expression and membrane cholesterol level in MSCs (Figs. 2–4). Consistent with previous studies performed on fibroblasts [15, 16], we observed that the membrane cholesterol level is regulated by CAV-1-mediated caveolar pathways in MSCs. Although the mechanism behind these actions is unknown, membrane cholesterol may regulate the CAV-1 transcription via modulating the phosphoinositide 3-kinase (PI3K)/Akt signaling pathways. It has been reported that MβCD-mediated cholesterol depletion in cells significantly inhibited the activation of Akt and further reduced PI3K activation, suggesting that the membrane cholesterol level may regulate the PI3K activity [41]. More recently, another study has reported that the PI3K/Akt signaling pathway may be directly involved in CAV-1 mRNA and protein expressions in embryonic stem cells [42]. Taken together, these findings suggest that membrane cholesterol may regulate the CAV-1 transcription level via modulating PI3K/Akt signaling in MSCs. Furthermore, our results suggest that caveolae and caveolin protein levels in the cell may determine lipid raft composition and organization by regulating membrane cholesterol content. Of note, our study was designed to investigate the effect of plasma membrane cholesterol alone by minimizing cholesterogenesis, the effect of intracellular cholesterol biosynthesis. Therefore, it is concluded that changes in membrane cholesterol and CAV-1 mRNA levels in MSCs after perturbation of cholesterol/CAV-1/caveolae homeostasis are not affected by changes in intracellular cholesterol levels.

Another interesting finding of this study is that interactions between cholesterol and CAV-1 protein may influence caveoliogenesis, the formation of morphologically identifiable caveolae. Treatment of cells with MβCD results in significantly lower caveolae content, while cholesterol enrichment results in significantly higher caveolae content (Fig. 4a, b), with concomitant upregulation of CAV-1 mRNA expression (Fig. 2a). In agreement with previous studies performed by others [38, 43], our results indicate that cholesterol may play a crucial role in caveolae development, although further investigation is required to determine the mechanistic steps responsible for this observation. To date, CAV-1 protein expression is generally assumed to be essential and sufficient for caveolae formation; however, previous studies [38, 43, 44] and results from the current investigation suggest that formation of stable caveolae may depend on the interactions between cholesterol and CAV-1 protein. It is also possible that cellular membrane cholesterol levels can function as a determinant of CAV-1 gene and caveolae abundance in MSCs.

Perturbation of cholesterol/CAV-1/caveolae homeostasis in MSCs also shows a profound effect on membrane rigidity/fluidity. Our findings showed that depletion or enrichment of membrane cholesterol significantly increased or decreased membrane fluidity, respectively. More importantly, si CAV-1-MSCs showed significantly increased membrane fluidity compared with si Ctrl-MSCs (Fig. 5). Taken together, these results confirm and ascertain that knockdown of CAV-1 mRNA in MSCs causes a reduction in cholesterol concentration in the plasma membrane. Importantly, these findings suggest that regulating CAV-1 mRNA levels in MSCs alone can be used to control cell membrane fluidity. Interestingly, siRNA transfection alone appears to have an impact on membrane fluidity as membrane fluidity was significantly lower in si Ctrl-MSCs compared with untreated MSCs (*p* < 0.0001; Fig. 5), most likely due to the presence of lipofectamine in cells during siRNA transfection. Exposing cells to a lipid-based transfection reagent may cause the transient increase in integration of lipid components to the cell membrane, leading to a reduction in membrane fluidity compared with untreated MSCs. Nevertheless, depletion of cholesterol or downregulation of the CAV-1 gene both independently decreased the membrane cholesterol level (Fig. 3) and increased membrane fluidity (Fig. 5) compared with their control counterparts.

The presence of cholesterol in the plasma membrane is important not only for membrane fluidity and structure, but also for cell adhesion. A possible role for cholesterol and membrane lipids in controlling cell morphology and motility has been previously reported [45–47]; however, their possible involvement in regulating cell adhesion is unexplored. A recent study demonstrates that the modification of cholesterol levels in MSC membranes causes significant changes in cell adhesion to CL and FN, which is directly related to tissue rigidity in humans [22, 25]. Acute depletion of cholesterol and CAV-1 mRNA in MSCs results in significantly reduced rates of cell adhesion, while cholesterol supplementation results in better cell adhesion (Fig. 6). Previous studies have addressed the factors that contribute to changes in the cell adhesion rate, including ECM structure and components, focal adhesion proteins, and actin cytoskeleton arrangements. Additionally, it has been shown that cholesterol enhances the adhesion of monocytes to the endothelium by translocating cell adhesion molecules—bound to caveolin-1—out of caveolae for more immediate cell-cell interaction [19]. Meanwhile, cholesterol depletion decreases L27 cell adhesion and migration to FN-coated substrates [47] and is known to decrease endothelial cell adhesion on polyacrylamide gel due to increases in the average traction force that cells exert on the substrate. These larger traction forces translate into cell traction activities, which further decrease monolayer stability, adhesion, and cell spreading [17]. In addition to the above, another important factor influencing cell adhesion is integrin expression [23].

Our results show that modification of cholesterol/CAV-1/caveolae homeostasis is closely related to changes in cell surface integrin levels; these changes are likely, at least in part, responsible for alterations in the cell adhesion rate on CL and FN. Our data demonstrate that the level of integrins per cell is significantly higher on Chol-MSCs compared with other groups (Fig. 7). These Chol-MSCs show higher adhesion rates compared with untreated or MβCD-MSCs (Fig. 6), suggesting that excess cholesterol loading on membranes increases both integrin expression level and cell adhesion rate. Integrins are major matrix-adhesive, mechanosensing receptors for cell substrates and are involved in MSC substrate rigidity-dependent responses [22, 25, 27]. Since cholesterol/CAV-1/caveolae perturbation affects adhesion processes in MSCs, mediated by changes in integrin availabilities, and the stability of integrin adhesion appears to be important for MSC substrate rigidity sensing, MSC substrate rigidity responses may be sensitive to membrane cholesterol.

Given the critical role of integrins in governing cell morphology [48], membrane cholesterol may therefore play a role in integrin-mediated cell shape changes. In fact, we observed here that changes in cell adhesion and integrin expression levels due to membrane cholesterol modification in MSCs were accompanied by remodeling of the actin cytoskeleton and changes in cell morphology (Fig. 3b). A reduction in membrane cholesterol resulted in rounding of the MSCs and disorganization of the actin networks, shown by phalloidin staining, while the Chol-MSC group were well spread and retained spindle shapes (Fig. 3b). Interestingly, changes in CAV-1/caveolae expression on MSC membranes had no significant effect on changes in cell shape. Further studies are required to better understand cholesterol-mediated cell morphology changes; however, our findings highlight the fact that cholesterol also affects cell morphology, which may be mediated by integrin activities on MSC membranes.

More importantly, overall results from our previous and current studies point out that alterations of biological activities in MSCs using cholesterol may be applied to the use of MSCs in regenerative medicine applications. Modified cell adhesion characteristics can be of relevance to the introduction of MSCs to a biomaterial surface for various types of tissue engineering applications. For example, cells for artificial heart valves or blood vessel engineering are often required to be nonadherent to biomaterials to avoid thrombosis and embolism [49]. Meanwhile, in skeletal muscle or bone tissue engineering, cells need to be adherent to materials used in scaffolds for subsequent proliferation, differentiation, and tissue reconstruction [50, 51]. Therefore, acute treatment of MSCs with either MβCD or free cholesterol can be a useful tool to adjust adhesion properties of MSCs to optimize the tissue engineering conditions. It has been reported that cholesterol may also affect the MSC differentiation potential, in particular osteogenic differentiation. Previously, we have reported that CAV-1 and cholesterol play an important role in regulating osteogenesis-driven PI3K/Akt signaling in MSCs [32]. Moreover, a study from Li et al. has reported that cholesterol loading enhances osteoblastic differentiation in mouse MSCs, possibly mediated by induction of BMP2 and RUNX2 expressions [52]. More interestingly, a study from Hamidouche et al. has shown that priming for β1 integrin expression in cells strongly enhanced osteogenesis [53]. Taken together, the results from our study additionally suggest that MSCs with increased membrane cholesterol content may respond more sensitively to osteogenic inductions, and therefore result in more efficient bone regeneration.

Recently, several studies have reported evidence that cholesterol-regulating drugs such as statins influence stem cell functions. The statins class of drugs lower the level of cholesterol in the blood by inhibiting the production of the enzyme in the liver that is responsible for cholesterol biosynthesis [54]. Previously, statins have been shown to have a significant beneficial effect on treating cardiovascular- and atherosclerosis-related diseases as well as increasing bone mass [55–57]; however, according to more recent studies, statin therapies may have detrimental effects on stem cell function. For example, Izadpanah and colleagues found that statins significantly reduced the growth rate of MSCs as well as the potential of MSCs to differentiate into macrophages [58]. Others have reported that long-term statin treatment strongly inhibited MSC differentiation into bone, cartilage, and smooth muscle cells, and induced higher apoptotic cell death [59, 60]. Results from our study also suggest that a decrease in membrane cholesterol may have negative effects on MSC behavior, particularly on adhesion properties and cytoskeletal organization (Figs. 3 and 6). Although the only report of its kind, a study has suggested that pravastatin therapies to lower cholesterol levels in hypercholesterolemic patients resulted in a significant reduction in erythrocyte and platelet membrane cholesterol content, which further caused changes in the Na^+^/K^+^ pump activity in these cells [61]. Whether these changes in membrane cholesterol and function observed during cholesterol lowering also occur in other cells is unknown. Taken together, these studies suggest that cholesterol lowering in stem cells, either by statin treatment or membrane cholesterol depletion, may impose negative effects on stem cell behaviors and activities. Therefore, in future studies, it will be interesting to investigate short- and long-term effects of statin treatments on membrane and intracellular cholesterol levels as well as effects of statins on MSC behavior and function.

## Conclusions

In conclusion, the two important findings of this study are: 1) membrane cholesterol and CAV-1/caveolae in human bone marrow-derived MSCs are directly and functionally linked, such that cholesterol level is regulated by CAV-1/caveolae; and 2) modification of cellular cholesterol/CAV-1/caveolae homeostasis in MSC membranes has significant effects on plasma membrane fluidity, cell adhesion, and cell surface integrin levels. Membrane cholesterol depletion in MSCs results in reductions in CAV-1 mRNA and protein expression, the number of caveolae, the adhesion rate to CL and FN, and cell surface integrin levels, and, in turn, causes increased membrane fluidity. Cholesterol enrichment has the exact opposite effects. These observations underscore the importance and possibility of cholesterol in controlling and regulating the biological activities of MSCs. In a broader context, since cholesterol is a major health concern particularly in the US and is easily and frequently managed, results from this study could be applied to treatments for diseases which involve cholesterol or lipid organization disorders and to manipulate biological activities of MSCs for cell therapy and/or tissue engineering, especially for bone tissue engineering.

## Additional file

Additional file 1: **Table S1.** Cell groups used in this study. Each cell group was generated by pooling MSCs from three to four donors. Donor MSCs are specified by age (number) and gender (m, male; f, female). (PDF 14 kb)

## Abbreviations

2D: Two-dimensional
APC: Allophycocyanin
BSA: Bovine serum albumin
CAV-1: Caveolin-1
Chol-MSC: Cholesterol-enriched mesenchymal stem cell
chol-MβCD: Cholesterol-methyl-β-cyclodextrin inclusion complexes
CL: Collagen I
CS-FBS: Charcoal stripped fetal bovine serum
DMEM: Dulbecco’s modified Eagle’s medium
ECM: Extracellular matrix
FACS: Fluorescence-activated cell sorting
FBS: Fetal bovine serum
FGF: Fibroblast growth factor
FN: Fibronectin
HRP: Horseradish peroxidase
ISM: Isolation medium
MSC: Mesenchymal stem cell
MTS: 3-(4,5-Dimethylthiazol-2-yl)-5-(3-carboxymethoxyphenyl)-2-(4-sulfophenyl)-2H-tetrazolium
MβCD: Methyl-β-cyclodextrin
MβCD-MSC: Cholesterol-depleted mesenchymal stem cell
PBS: Phosphate-buffered saline
PCR: Polymerase chain reaction
PE: Phycoerythrin
PFA: Paraformaldehyde
PI3K: Phosphoinositide 3-kinase
PL: Plain cell culture plastic
PM: Proliferation medium
PVDF: Polyvinylidene fluoride
si CAV-1-MSC: Mesenchymal stem cell transfected with caveolin-1 small interfering RNA
si Ctrl-MSC: Mesenchymal stem cell transfected with control, nonspecific small interfering RNA
TBST: Tris-buffered saline + 0.05% Tween 20
