# Supplementary material for: Influence of cholesterol/caveolin-1/caveolae homeostasis on membrane properties and substrate adhesion characteristics of adult human mesenchymal stem cells
Source: Stem Cell Res Ther. 2018 Apr 3;9:86. doi: 10.1186/s13287-018-0830-4 (PMC5883280; doi:10.1186/s13287-018-0830-4)
Supplement: Supplementary file 1 — Table S1. Cell groups used in this study. Each cell group was generated by pooling MSCs from three to four donors. Donor MSCs are specified by age (number) and gender (m, male; f, female). (PDF 14 kb) [file 13287_2018_830_MOESM1_ESM.pdf]

Additional file1. Cell groups used in this study. Related to Figures 1-7.

Group	Donor MSCs
1	57m, 61m, 58m
2	47f, 62f, 54f
3	62m, 65m, 58f, 66f

Additional file 1. Cell groups used in this study. Each cell group was generated by pooling MSCs from 3-4 donors. Donor MSCs are specified by age (number) and gender (m, male; f, female).
